# Elevated glucose increases *Staphylococcus aureus* antibiotic resistance in a cystic fibrosis airway epithelial cell infection model

**DOI:** 10.1128/iai.00178-25

**Published:** 2025-09-22

**Authors:** Emily M. Hughes, Meghan J. Hirsch, Joshua T. Huffines, Stefanie Krick, Megan R. Kiedrowski

**Affiliations:** 1 Division of Pulmonary, Allergy and Critical Care, Department of Medicine, The University of Alabama at Birmingham 654677 , Birmingham, Alabama, USA; St Jude Children's Research Hospital, Memphis, Tennessee, USA

**Keywords:** antibiotic resistance, bacterial aggregation, biofilm, CF-related diabetes, *Staphylococcus aureus*, cystic fibrosis

## Abstract

In a healthy lung, the airway epithelium regulates glucose transport to maintain low glucose concentrations in the airway surface liquid (ASL). However, hyperglycemia and chronic lung diseases, such as cystic fibrosis (CF), can result in increased glucose in bronchial aspirates. People with CF are also at increased risk of lung infections caused by bacterial pathogens, including methicillin-resistant *Staphylococcus aureus*. Yet, it is not known how increased airway glucose availability affects bacteria in chronic CF lung infections or impacts treatment outcomes. To model the CF airways, we cultured immortalized CF (CFBE41o-) and non-CF (16HBE) human bronchial epithelial cells at the air-liquid interface (ALI). Glucose concentrations in the basolateral media were maintained at 5.5 or 12.5 mM to mimic a normal and hyperglycemic milieu, respectively. We found that glucose concentrations in the ASL of ALI cultures maintained in normal or high glucose mimicked levels measured in breath condensate assays from people with CF and hyperglycemia. Additionally, we found hyperglycemia increased *S. aureus* aggregation and antibiotic resistance during infection of cells maintained in high glucose compared to normal glucose conditions. Heightened antibiotic resistance was not observed during *in vitro* growth with elevated glucose. Limiting glucose with 2-deoxyglucose both decreased aggregation and reduced antibiotic resistance back to levels comparable to non-hyperglycemic conditions. These data indicate that hyperglycemia alters *S. aureus* growth during infection and may reduce efficacy of antibiotic treatment. Glucose restriction is a potential option that could be explored to limit bacterial growth and improve treatment outcomes in chronic airway infections.

## INTRODUCTION

Cystic fibrosis (CF) is a rare genetic disease that affects multiple organs in the body, including the lungs. Mutations in the cystic fibrosis transmembrane conductance regulator (CFTR) gene result in improper ion transport, resulting in decreased mucociliary clearance and accumulation of thick and sticky mucus on epithelial surfaces (1, 2). In the lungs, abnormal mucus traps bacterial pathogens and provides an environment that harbors persistent infection because of mucus that cannot be properly cleared from the lungs, resulting in chronic inflammation (2, 3). In CF, the pancreas is also affected by a mucus buildup that causes cellular damage and scarring, preventing insulin from being made while also creating a physical barrier preventing insulin from being properly secreted (4, 5). Additionally, there is often pancreatic remodeling leading to the loss of beta cells, reducing the amount of insulin being produced. This results in the onset of CF-related diabetes (CFRD) in approximately 30% of the US CF population (6, 7).

*Staphylococcus aureus* is the most common pathogen isolated from the CF lung. Even with the introduction of highly effective modulator therapy (HEMT), people with CF (pwCF) still have very high rates of positive *S. aureus* cultures, with around 60% of the US CF population having at least one *S*. *aureus* positive culture in 2023 (7–9). Methicillin-resistant *S. aureus* (MRSA) is a continued health concern in chronic respiratory diseases, including CF, due to antibiotic resistance (10, 11). Studies in non-CF populations have shown that having diabetes increases the risk of health care-associated pneumonia caused by MRSA, while other studies have shown that ICU patients who are intubated and have ≥1 mM of glucose in their bronchial aspirates had significantly more staphylococcus species present, including significantly more MRSA present (12–14). This correlation is of particular importance to pwCF because HEMT has only modestly reduced the rates of CFRD, and insulin is currently the only treatment option for maintaining blood glucose levels (15–18). CFRD is a significant risk factor for developing persistent MRSA infections, and MRSA and CFRD have been shown to lead to worse outcomes than either factor alone (19, 20).

It has been hypothesized that glucose restriction is one factor that helps to keep the airway sterile by limiting nutritional availability for microbes that colonize the respiratory tract or prevent the invasion by pathogens, such as *S. aureus*. A healthy lung epithelium tightly regulates glucose homeostasis, keeping free glucose concentrations well below 1 mM in the airway (21, 22). Hyperglycemia disrupts this homeostasis, increasing airway glucose concentrations up to 1.89 mM. Chronic lung diseases, like CF, further disrupt glucose homeostasis, with pwCF found to have up to 3.13 mM glucose in bronchial aspirates (22–24). CFRD causes a further increase, resulting in up to 6.07 mM of available glucose in the lung (22).

Despite the correlation between diabetes and *S. aureus* lung infections, very few studies have examined how the diabetic lung environment and increase in airway glucose availability impact *S. aureus* infections or how these conditions might affect antibiotic treatment outcomes. In this study, we use human bronchial epithelial cells cultured in elevated glucose conditions to mimic the normal and hyperglycemic lung environments. Using this model, we found that the hyperglycemic lung environment significantly decreases the *S. aureus* response to antibiotic treatment, and limiting glucose availability at the airway surface can reverse this effect.

## RESULTS

### A hyperglycemic air-liquid interface cell culture model accurately replicates glucose levels measured in the human lung

**Fig 1:**
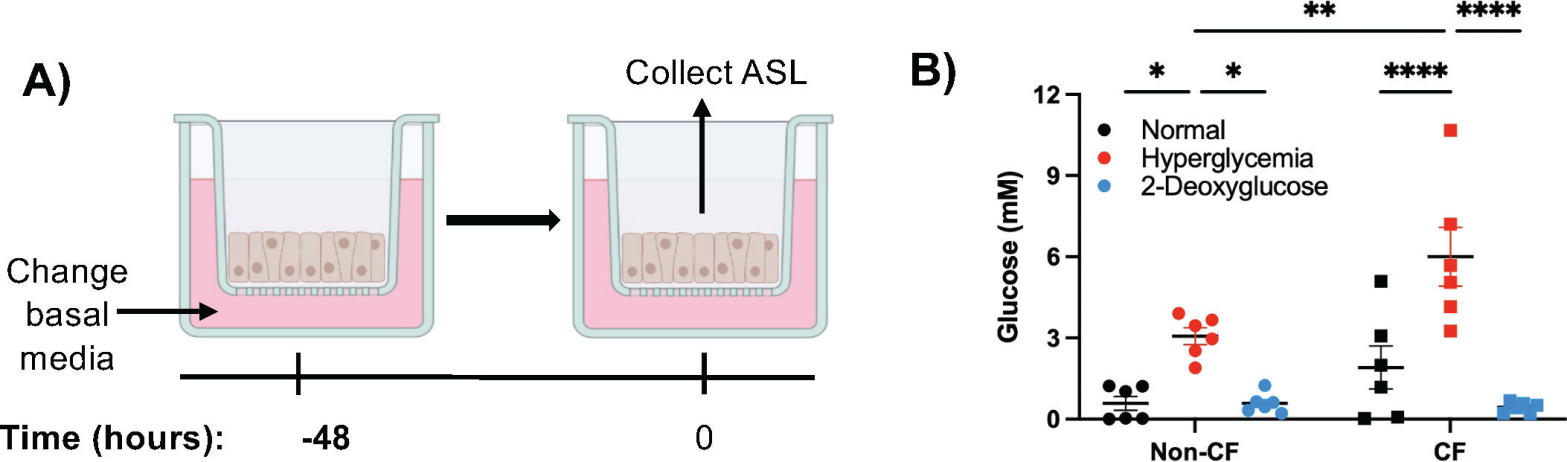
Hyperglycemic conditions alter glucose concentrations in the airway surface liquid (ASL). (**A**) Cells at the air-liquid interface (ALI) were switched to different glucose media 48 h prior to infection, RNA preparation, or ASL collection. (**B**) ASL glucose measurements from non-CF (circles) and CF (squares) ALI cultures. Data reported as mean ± SEM. *P < 0.05, **P < 0.01, and ****P < 0.0001.

To determine the impact glucose has on *S. aureus*, we first characterized our cell culture model using cells cultured at air-liquid interface (ALI). The physiologically relevant glucose conditions of 5.5 mM are used to represent normal blood glucose levels, while 12.5 mM is used to represent a hyperglycemic blood glucose level. After establishing our cell culture conditions, we then measured the amount of glucose found in the airway surface liquid (ASL) (Fig. 1A). We found that our model closely replicates ASL glucose concentrations measured in clinical samples (Fig. 1B) (22). We then wanted to determine if we could limit the glucose in the ASL. To accomplish this, we used a competitive inhibitor, 2-deoxyglucose (2DG) (25). Using this, we found that it significantly lowered glucose levels in the ASL (Fig. 1B). To further characterize our model, and because 2DG inhibits glycolysis, which can cause cell death, we verified that the cell monolayer was still intact using transepithelial electrical resistance (TEER) (Fig. S1A). This also confirmed that the increase in ASL glucose is not due to a disruption in the tight junction integrity. We also determined no significant increase in lactate dehydrogenase (LDH), indicating that there is no increase in cellular cytotoxicity (Fig. S1B). Together, from these experiments, we can conclude that our model accurately represents the glucose conditions found in the lung and that hyperglycemia and the competitive inhibitor 2DG do not have a negative impact on our model.

### Short-term, high-glucose ALI culture of immortalized bronchial epithelial cells does not alter inflammatory cytokine levels

**Fig 2:**
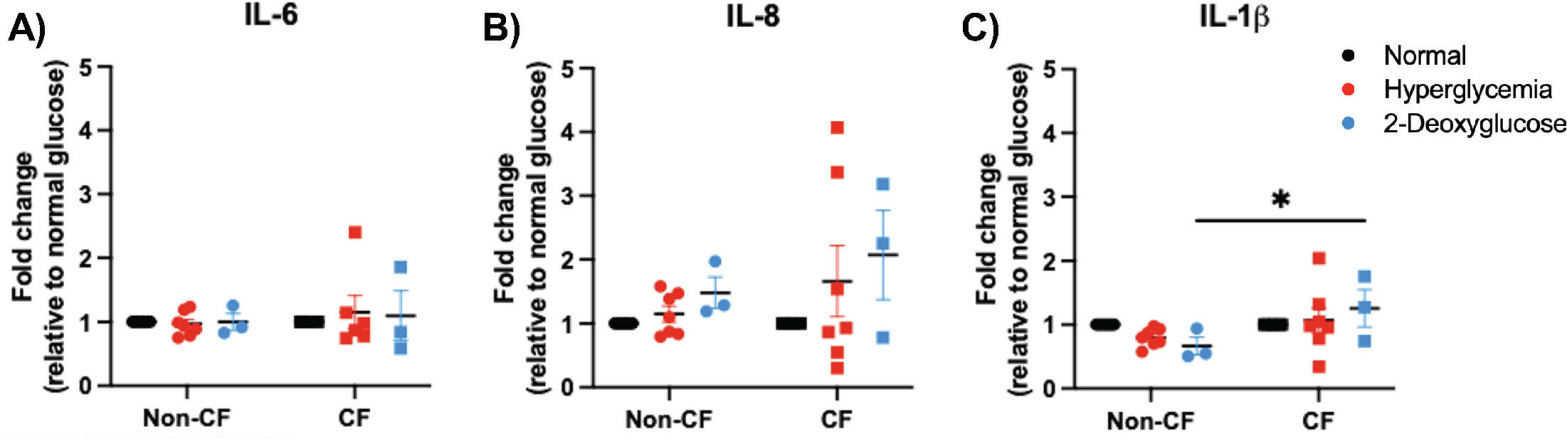
Hyperglycemia does not meaningfully impact pro-inflammatory cytokine levels. (**A**) IL-6 transcript and (**B**) IL-8 transcript, and (**C**) IL-1β transcript levels. Data reported as mean ± SEM. *P < 0.05.

Because individuals with hyperglycemia and CF are known to have increased systemic inflammation, we wanted to confirm if glucose levels affect the pro-inflammation cytokine expression in this model (26–28). Therefore, we measured the expression of common pro-inflammatory cytokines, IL-6, IL-8, and IL-1β, via RT qPCR (Fig. 2A through C). We observed that hyperglycemia did not significantly elevate inflammatory cytokines in either non-CF or CF cells compared to normal controls. There was an increase in IL-1β in CF cells treated with 2-DG compared to non-CF cells, indicating there may be some additional stress on CF cells with 2-DG treatment (Fig. 2C). IL-1β levels were found to be significantly increased in CF cells compared to non-CF cells in all treatment groups, including normal, hyperglycemic, and 2-DG treated conditions (Fig. S2). This is expected since the CF airways are known to have heightened IL-1β compared to the healthy airway (29). However, there was no significant difference in IL-β levels measured between CF groups. These data demonstrate that hyperglycemia alone is not significantly increasing inflammation in our model.

### *S.* aureus aggregate size is increased in CF hyperglycemic conditions

**Fig 3:**
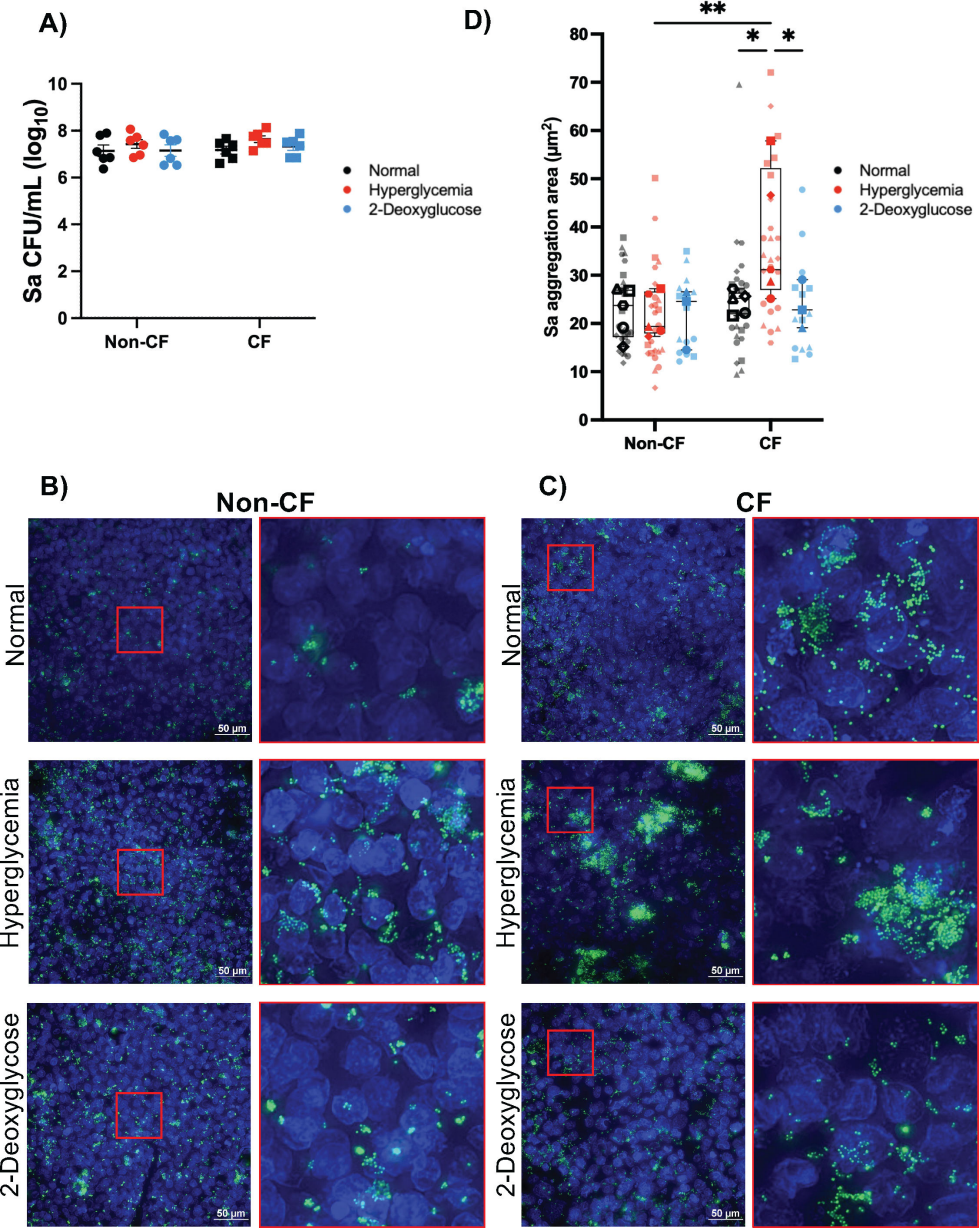
Hyperglycemic airway environment alters the *S. aureus* aggregation. *S. aureus* grown on non-CF or CF ALI cultures for 6 h. (**A**) *S. aureus* (Sa) bacterial burden in colony-forming units/mL (CFU/mL). Data reported as mean ± SEM. Widefield fluorescence microscopy images of GFP-expressing *S. aureus* (green) on non-CF (**B**) and CF (**C**) human bronchial cells (Hoechst, blue). (**D**) Quantification of the aggregation area for *S. aureus* aggregates 5 µm or larger in size. Independent biological replicates are indicated by data points with different shapes; averages for biological replicates are shown in solid color, with technical replicates indicated in transparent colors. Data reported as box and whisker plot min to max. Statistical analysis performed on biological replicates for all samples. *P < 0.05, **P < 0.01.

After confirming that our model replicates clinical data and that we can control ASL glucose levels, we then infected our ALI model with *S. aureus* USA100, a hospital-acquired strain of MRSA. We determined that there is no difference in *S. aureus* burden after 6 h on non-CF and CF cells cultured in normal or hyperglycemic conditions, and 2DG treatment does not significantly affect the total bacterial burden (Fig. 3A). However, imaging revealed a significant increase in the number of bacterial aggregates measuring over 5 µm in CF hyperglycemic conditions but not in non-CF hyperglycemic conditions (Fig. 3D). This cut-off was chosen, as it has been previously determined that this is the average size of bacterial isolates from the airways of people with CF (30, 31). The addition of 2DG reduced the aggregate size back to normal glucose conditions (Fig. 3B and C).

### Antibiotic resistance is increased in the ALI co-culture in hyperglycemic conditions

**Fig 4:**
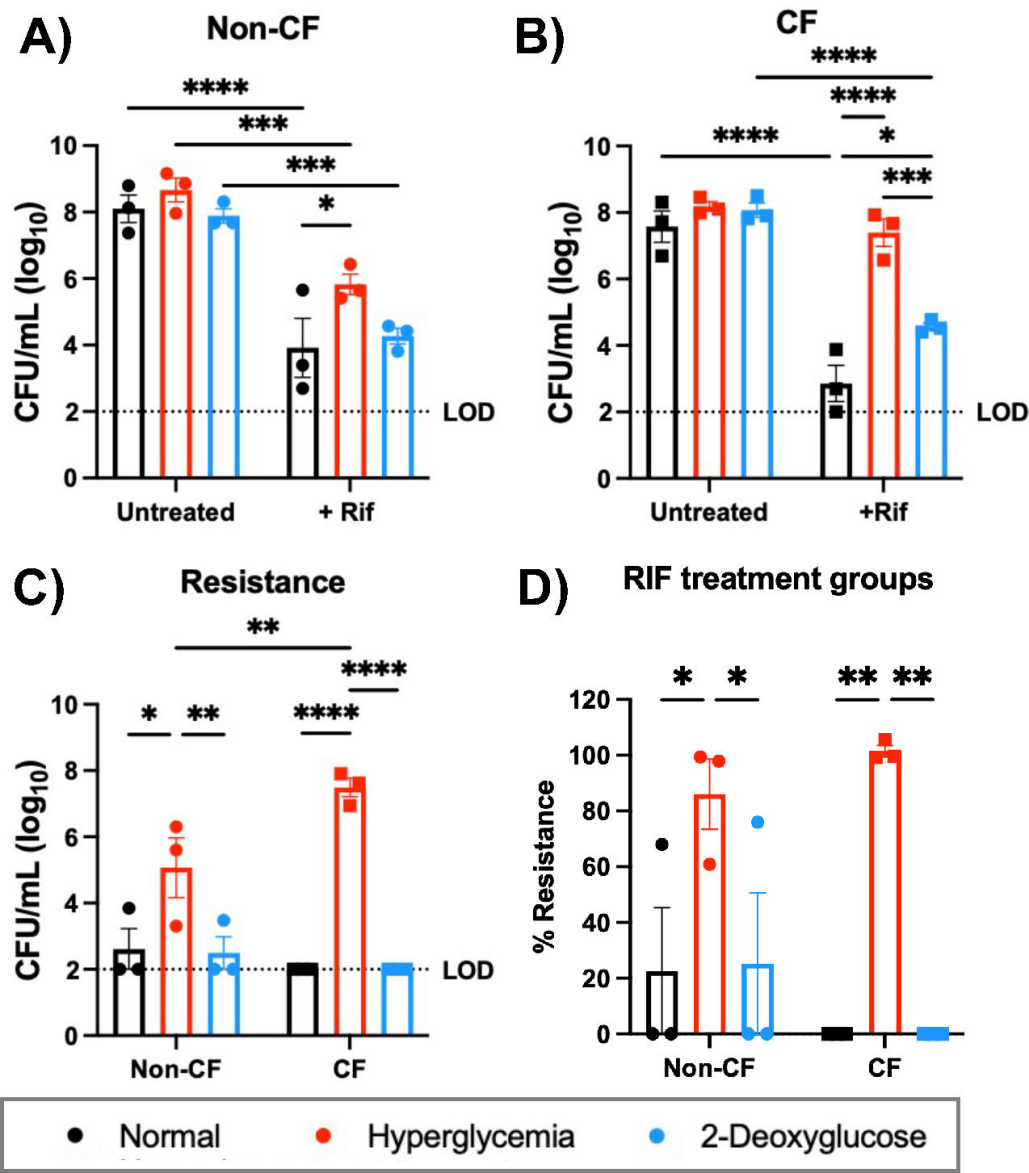
Hyperglycemic conditions increase *S. aureus* rifampicin resistance when co-cultured with human bronchial cells. CFUs of USA100 co-cultured with ALI cultures in the presence and absence of rifampicin treatment. (**A**) Non-CF cells. (**B**) CF cells. (**C**) Resistance determined in non-CF (circles) and CF (squares) co-culture samples. (**D**) Percent resistance in *S. aureus* populations for RIF-treated conditions. A horizontal line indicates the limit of detection (LOD). Data reported as mean ± SEM. *P < 0.05, **P < 0.01, ***P < 0.001, and ****P < 0.0001.

After observing that elevated glucose increases bacterial aggregation in our ALI model, we next wanted to determine the effects of hyperglycemia on the outcome of antibiotic treatment. To mimic an antibiotic intervention, we infected normal or hyperglycemic ALI cultures with *S. aureus* for 6 h to allow biofilms to establish and mimic an existing infection before adding rifampicin (RIF) and allowing the infection to proceed for an additional 24 h. We found that in the non-CF cells, antibiotic treatment significantly reduced the *S. aureus* burden, regardless of the glucose condition (Fig. 4A). Similarly, with CF cells, we found that in normal glucose conditions, RIF reduced bacterial burden to almost undetectable levels. However, culturing cells in hyperglycemic conditions completely negated the effects of antibiotic treatment, with no difference in the total *S. aureus* burden between antibiotic-treated and untreated conditions (Fig. 4B). To determine if RIF resistance was developing in *S. aureus*, leading to the differences we observed, we also measured the number of resistant bacteria at the final timepoint by enumerating colonies that grew on culture media containing rifampicin. We found that there were significantly more resistant bacteria in hyperglycemic samples treated with antibiotic than in the normal conditions also with antibiotic treatment (Fig. 4C). Surprisingly, this was not dependent on the cell type. The bacterial populations on non-CF cells and CF cells developed resistance at similar frequencies when exposed to the antibiotic despite RIF still being effective at reducing the overall bacterial burden on the non-CF cells (Fig. 4D). Bacterial resistance was not observed in inoculums or at the 6 h time point in either condition (data not shown). To confirm that this was a glucose-dependent response, we again utilized 2DG to limit ASL glucose. 2DG treatment significantly decreased the number of resistant *S. aureus* colonies, and the total burden of RIF-resistant *S. aureus* resembled the burden observed in normal glucose conditions for both cell types (Fig. 4A through D).

### Elevating glucose *in vitro* culture does not result in increased antibiotic resistance

**Fig 5:**
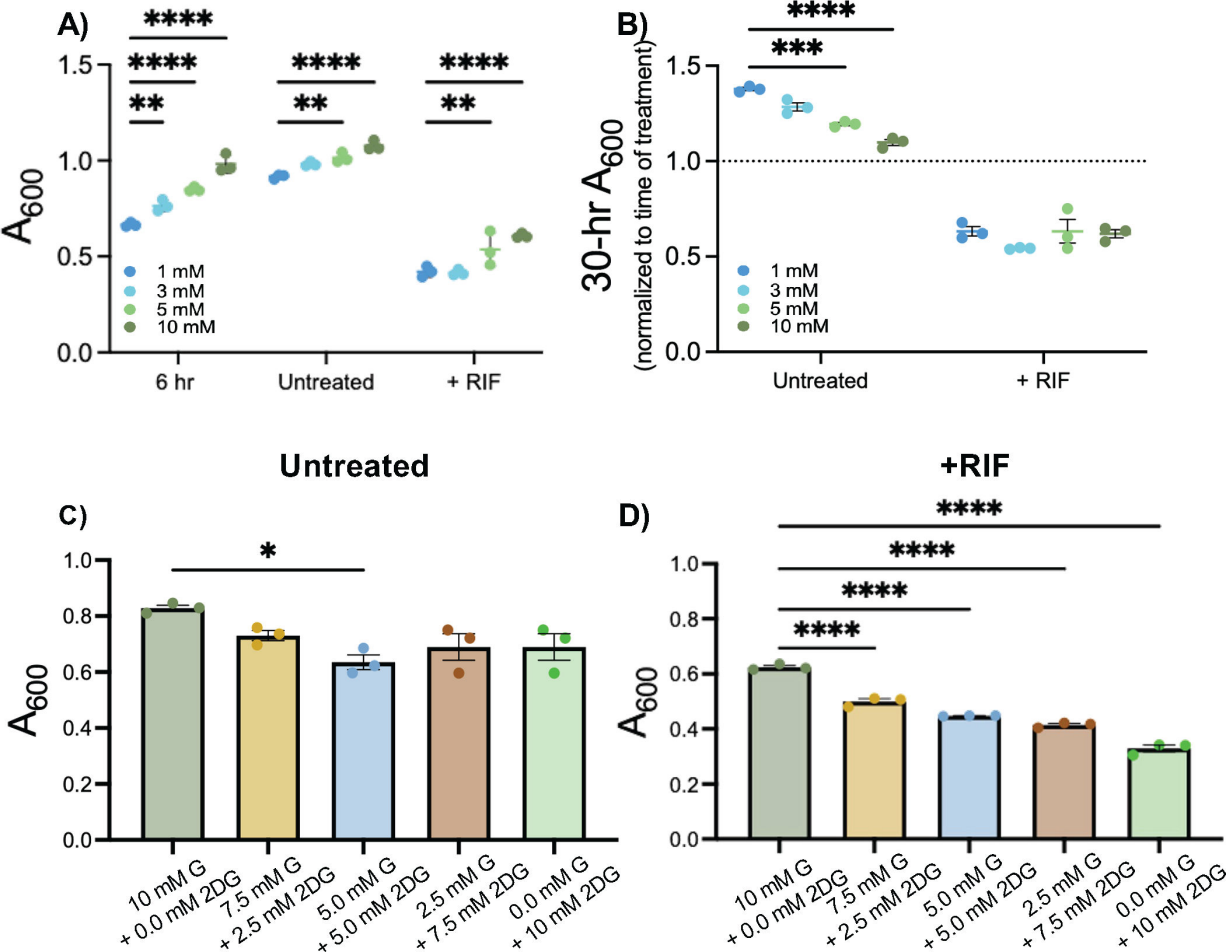
2-Deoxyglucose enhances the rifampicin-mediated killing of *S. aureus*. (**A**) A_600_ value of *S. aureus* measured at 6 or 30 h with or without rifampicin (RIF) treatment in the presence of increasing concentrations of glucose. (**B**) Final time point (30 h) A_600_ normalized to A_600_ measured at the time of RIF addition (6 h). (**C**) Final A_600_ of *S. aureus* USA100 in tryptic soy broth with glucose (G) and 2-deoxyglucose (2DG) after 30 h of growth. (**D**) Final A_600_ of *S. aureus* USA00 in rich media with glucose (G) and 2DG with RIF added after 6 h of growth. Final time point after 30 h total growth. Data reported as mean ± SEM. *P < 0.05, **P < 0.001, ***P < 0.001, and ****P < 0.0001.

After seeing the differences in bacterial aggregation and antibiotic resistance resulting from elevated glucose in our cell culture model, we next asked if these effects could be mimicked in broth culture in the absence of airway epithelial cells. To do this, we utilized the rich culture media commonly used for the growth of *S. aureus*, tryptic soy broth (TSB), and defined media replicating the nutrients available in the CF lung environment, synthetic CF media (SCFM) (32). We prepared base media lacking glucose and added defined amounts of glucose to achieve final concentrations that mimicked levels measured in ASL of non-CF and CF cells cultured in normal or hyperglycemic conditions. Using this range of glucose concentrations, we found that in both rich media and defined media, the addition of RIF was able to prevent growth of *S. aureus* when added concurrently with the bacterial inoculum (Fig. S3A and 4A). We next grew *S. aureus* for 6 h before adding RIF to mimic an existing infection as tested in the ALI co-infections. In TSB, we observed similar results as with ALI infections, where RIF was less effective at killing *S. aureus* in high glucose conditions (Fig. 5A; Fig. S3B). Here, unlike in the ALI infections, only one replicate of the 5 mM glucose developed resistance (Fig. S3B). This was not observed in other glucose concentrations and was most likely due to the fact that spontaneous RIF resistance can occur (33). We also observed increased growth in the higher glucose concentrations at the time of RIF addition, leading to an overall higher growth at the final timepoint (Fig. 5A; Fig. S3B). This initial increased growth accounts for the seemingly less effective RIF treatment at the higher glucose concentrations. When the final treatment absorbances are normalized to the respective absorbances at the time treatment was added, there is no difference in values (Fig. 5B), indicating that RIF treatment is equally effective, regardless of the glucose concentration. RIF was also effective at killing *S. aureus* in SCFM; however, here the 1, 5, and 10 mM conditions each had one replicate develop resistance (Fig. S4).

Because *S. aureus* is known to utilize glucose rapidly in culture, we determined that by the 6 h time point, glucose had been depleted in TSB conditions (Fig. S5A). To determine if glucose depletion was contributing to the lack of development of RIF resistance, we supplemented TSB cultures with additional glucose concurrently to the addition of RIF at 6 h and measured the endpoint resistance (Fig. S5B). We found that RIF resistance did not develop more frequently when glucose was added back to cultures (Fig. S5C). We observed low levels of resistance in *S. aureus* populations in all conditions exposed to RIF and low-level development of resistance with the addition of 1 and 3 mM glucose. A lower frequency of resistance was observed compared to what we consistently found in the hyperglycemic ALI co-culture model, indicating that the host metabolism in high glucose environments likely plays a yet undetermined role in promoting the development of resistance in *S. aureus*.

Additionally, culturing *S. aureus* in media containing glucose and 2DG did not adversely affect growth (Fig. 5C). Furthermore, RIF treatment added after 6 h of growth was significantly more effective in conditions with glucose and 2-deoxyglucose than with just glucose alone (Fig. 5D). These results indicate that allowing *S. aureus* to establish a biofilm before antibiotic exposure may increase the risk of antibiotic resistance developing, while limiting glucose may be an effective strategy to increase antibiotic effectiveness. Overall, we did not find that elevated glucose in either TSB or SCFM promoted the development of RIF resistance to similar levels as observed in the airway cell co-culture, suggesting that airway epithelial physiology in hyperglycemic conditions may result in altered host-pathogen interactions leading to reduced antibiotic effectiveness.

## DISCUSSION

In this study, we established an air-liquid interface cell culture hyperglycemia model for the CF airway that replicates the elevated glucose levels previously reported from the lung of those with diabetes and CF (22). We found no adverse effects to the cells due to exposure to elevated glucose in growth media and no increase in inflammatory cytokines between non-CF and CF cell types. However, we did find glucose-dependent differences, with CF cells showing elevated baseline ASL glucose concentrations that further increased upon hyperglycemic culture. Other studies have cultured cells in hyperglycemic conditions for 1 to 3 weeks and have shown varying results in changes to the cell monolayer integrity as measured by TEER (34, 35). Additionally, Bengtson et al. found that non-CF primary bronchial epithelial cells cultured at 12.5 mM glucose for 3 weeks had increases in IL-6, IL-8, and IL-1β mRNA, while primary CF cells did not have the same increase in mRNA (34). Our model exposed immortalized cells to high glucose for a shorter time period, and testing prolonged exposure to hyperglycemic conditions may result in similar increases in inflammation as observed in these studies.

Despite not observing a difference in the overall *S. aureus* burden on cells cultured in hyperglycemic conditions, we saw that *S. aureus* formed larger aggregates on CF cells cultured in high glucose than in other conditions. The persistent infections driven by the formation of biofilm-like aggregates are a hallmark of chronic CF airway infections. Yet, the increased aggregate size we observed on CF cells cultured at high glucose is surprising because it has been shown that glucose can disperse the *S. aureus* biofilm through the inhibition of *agr*. Our results could be explained by the difference in the glucose concentrations. The highest amount of glucose in our study was 10 mM, while studies showing glucose-induced dispersal were two to 10 times that amount (36, 37). Additionally, it has been shown that it is not actually glucose that is causing the dispersal, but rather the low-acidity environment generated by the *S. aureus* utilization of glucose (38). It has been shown that pH is lower in the CF airways than in the non-CF airways due to the dysfunction in the CF transmembrane conductance regulator channel (39). Exploring if hyperglycemia further contributes to pH imbalance in the CF airway warrants further investigation through testing of clinical samples from individuals with CFRD or hyperglycemia.

Our finding that elevated ASL glucose results in different outcomes to antibiotic treatment may help explain why people with increased airway glucose are more susceptible to persistent *S. aureus* lung infections. Additionally, a recent study by Shook et al. observed similar antibiotic outcomes in a diabetic wound model. The lack of innate immune cells in our model confirms that the development of resistance is driven by a mechanism other than the host immune response, confirming findings by Shook et al. (40). Our data showing that glucose limitation with 2-DG in the ALI hyperglycemia model prevented the emergence of RIF-resistant *S. aureus* also agree with Shook et al.’s observation that controlling the blood sugar in the diabetic mouse model decreased the incidence of antibiotic resistance. Our results indicate that increasing glucose in TSB and SCFM was not sufficient to increase resistance to levels observed in the hyperglycemic ALI model and that the host is playing a yet undefined role in the development of resistance.

Future studies in our hyperglycemic ALI co-infection culture model can address how HEMT affects glucose availability at the airway epithelial interface and infection outcomes. Currently, there have been no clinical studies to determine how HEMT alters the amount of glucose found in the lungs of pwCF or how the bacteria present in the lung are changing with the restoration of the CFTR activity. If it is determined that HEMT does not correct elevated lung glucose levels, one potential treatment strategy could be augmenting antibiotic treatments with a glucose restriction therapy like 2-deoxyglucose, as our model showed no adverse effects with short-term use and effective reduction in bacterial loads.

There are some limitations to our model, including a lack of innate immune cells, which could have an altered response in hyperglycemic conditions and could exacerbate inflammatory cytokines. Studies with primary cell lines have shown increased cytokine levels in response to hyperglycemia, albeit for a much longer exposure time. The use of primary cell ALI cultures would allow us to address the question of how hyperglycemia is affecting ciliary beat and mucociliary clearance, both of which are important for clearing airway mucus and preventing infections. We also know that many infections in CF are not isolated to one species of bacteria but are polymicrobial in nature and even include viral infections. Not only do we not know if other bacteria can benefit from the increase in airway glucose, but we also do not know how their presence is affecting *S. aureus* in these elevated glucose conditions. Furthermore, it is known that viral infections can alter the host's metabolism, so a further exploration of how virus-induced metabolic changes are altering the lung environment and if they are altering glucose availability in airway is an important consideration for future studies.

## MATERIALS AND METHODS

### Bacterial strains and growth conditions

A *Staphylococcus aureus* USA100 strain representative of the MRSA isolates commonly observed in CF was used for these studies (41). *S. aureus* with or without plasmid pCM29 encoding green fluorescent protein (GFP) (42) was cultured overnight at 37°C with shaking in tryptic soy broth (TSB; BD Bioscience). Overnight cultures were inoculated using a single colony grown on TSB with 1.5% agar (TSA, BD Bioscience) at 37°C. Bacterial growth curves were done using TSB without dextrose (BD Bioscience) supplemented with glucose (Gibco) and/or 2-deoxyglucose (2DG, EMD Millipore Sigma) to achieve final concentrations. Approximately 1 × 10^6^ bacteria were inoculated per well in 96-well plates. Growth curves were run for 30 h in total in a Tecan Spark automated multimode microplate reader. If antibiotic was added after 6 h of growth, the microplate reader was paused, and rifampicin (Fisher BioReagents) was added to equal a final concentration of 35 µg/mL per well.

A modified synthetic cystic fibrosis medium (SCFM) was made according to Palmer et al., with glucose added to achieve 1, 3, 5, or 10 mM final concentrations (32). When antibiotic was added at the time of inoculation, bacterial colony-forming units (CFUs) were determined at the time of inoculum and at the assay endpoint. Bacteria were plated to both antibiotic-free TSA plates and TSA with rifampicin (10 µg/mL) to determine the total bacterial burden and the burden of rifampicin-resistant bacteria. For assays where antibiotic was added after 6 h of growth, CFUs were determined for the inoculum at the time of antibiotic addition (6 h), and endpoint (24 h) CFUs were measured by plating to TSA plates with and without rifampicin.

### Cell culture

#### Maintenance and ALI culture

Both non-CF (16HBE)- and CF (CFBE41o)-immortalized human bronchial epithelial cells were maintained as previously described (41) in minimal essential medium (MEM; Gibco) supplemented with 10% fetal bovine serum (FBS; Gibco) and pen-strep. Briefly, air-liquid interface cultures were established by seeding cells on transwell filters pre-coated with vitrogen plating media (VPM). After 1 week, media were removed from the apical side of the transwell, and cells were cultured at the air-liquid interface for at least 1 week before use in downstream assays. Cells were cultured at normal glucose (5.5 mM) until 24 or 48 h before use. At that time, ALI cultures were washed with MEM lacking serum and phenol red (Gibco) to remove any residual antibiotics. ALI cultures were then fed with antibiotic-free media with normal glucose (5.5 mM), hyperglycemic glucose (12.5 mM), or 2DG (7 mM 2DG + 5.5 mM glucose). Hyperglycemic media were made by adding glucose to the base MEM cell culture media. MEM containing 2DG (EMD Millipore Sigma) was made by adding 2DG to base MEM for a final concentration of 7 mM 2DG and 5.5 mM glucose.

For the ASL collection, 24 h after media change, 250 µL of clear, FBS-free MEM was added to the apical side of the ALI cultures grown in 12 mm transwell inserts and incubated for 24 h. The apical media were then collected, and glucose concentrations were determined using a glucose assay kit (Abcam). Transepithelial electrical resistance (TEER) was also measured after 6 and 24 h post-addition of clear MEM to ALI cultures using an EVOM2 epithelial Volt/Ohm meter (World Precision Instruments). Additionally, at the 24 h timepoint, basolateral media were collected to measure the lactate dehydrogenase release (LDH, Promega). Furthermore, some ALI samples were washed twice with ice-cold PBS++ (Gibco) and stored at −80°C for subsequent RNA extraction.

#### Co-culture infection assay

Subsequently, 24 h after changing the basolateral media of the ALI cultures to different glucose conditions as described above, the ALI cultures were infected with 1 × 10^6^ CFUs of live bacteria. After 1 h, the media containing any unattached bacteria were removed. CFUs were determined by the addition of 0.1% Triton (Bio-Rad) to the infected ALI samples, scraping total cells and bacteria, and plating serial dilutions on plain TSA or TSA with rifampicin (10 µg/mL) to enumerate total and antibiotic-resistant *S. aureus* populations (43). At 6 h post-inoculation, some infected ALI cultures were harvested for CFUs or imaging. At 6 h, some ALI cultures were treated with antibiotic (rifampicin 35 µg/mL) or vehicle control (MEM) added to the apical surface, and infection was allowed to proceed for an additional 24 h. After 24 h of treatment, final end-point CFUs were determined as described above.

### Fluorescence microscopy and biomass measurements

After 6 h of co-infection described above, the ALI cultures were fixed in 4% paraformaldehyde (PFA, Electron Microscopy Sciences). After overnight fixation, transwell filters were washed twice with PBS (Gibco), and then stained with Hoechst 33342 stain (Invitrogen). Filters were then cut out from transwell inserts using a razor blade and mounted on a microscope slide with Prolong Gold (Invitrogen). After drying, the filters were imaged on a widefield Ti Eclipse widefield fluorescence microscope (Nikon). After imaging, quantification of biofilms was done using the Nikon NIS-Elements AR software package (Version 5.42.02, Build 1801). Volume measurements were obtained for each image stack after automatic thresholding was performed in NIS-Elements AR. The NIS-Elements object count function was used to determine the number and the area of bacterial aggregates. Data analysis for bacterial aggregates was performed using RStudio version 2024.09.0, Build 375 “Cranberry Hibiscus” release (Posit Software). Aggregates with an area value less than 5 µm in size were excluded to eliminate noise and single cells from the data analysis (30, 31). Images shown and biomass measurements are representative of at least three independent experiments with at least five individual fields of view measured for each sample.

### RNA extraction and cytokine measurements

After treatments with glucose and/or *S. aureus*, total RNA was isolated, and RT-qPCR was run as previously described (44). Briefly, RNA was isolated using the GeneJET Purification Kit (Thermo Scientific). The RNA concentrations were assessed using NanoDrop, and cDNA was synthesized using Maxima H Minus cDNA Synthesis Master Mix (Thermo Fisher). RT-qPCR was performed on Applied Biosystems StepOnePlus using TaqMan primers interleukin 1-β (Hs01555410_m1, IL-1β), interleukin-6 (Hs00174131_m1, IL-6), and interleukin-8 (Hs00174103_m1, CXCL8) and reference gene glyceraldehyde 3-phosphate dehydrogenase (GAPDH). The relative difference in transcript levels was calculated using the ΔΔCt method with GAPDH as a reference gene.

Additionally, the secreted IL-1β, IL-6, and IL-8 protein levels were measured after treatments with glucose and/or *S. aureus* by enzyme-linked immunosorbent assays (ELISAs) (Invitrogen). These were performed according to the manufacturer’s protocol with the following assay sensitivities: human IL-1β (0.16–10 pg/mL), human IL-6 (2–200 pg/mL), and human IL-8 ELISA (2–250 pg/mL).

### Statistical analysis

Statistical analyses were performed with GraphPad Prism version 10.4.0 software (GraphPad by Dotmatics). One- or two-way analysis of variance (ANOVA) was determined as appropriate to measure the statistical differences between cell types and glucose conditions. Tukey’s post-hoc test was performed on multiple comparisons. *P* values were considered significant if less than 0.05.

## SUPPLEMENTAL MATERIAL

Supplemental material iai.00178-25-s0001.pdf Fig. S1 to S5.

ASM does not own the copyrights to Supplemental Material that may be linked to, or accessed through, an article. The authors have granted ASM a non-exclusive, world-wide license to publish the Supplemental Material files. Please contact the corresponding author directly for reuse.
