# Supplementary material for: Elevated glucose increases Staphylococcus aureus antibiotic resistance in a cystic fibrosis airway epithelial cell infection model
Source: Infect Immun. 2025 Sep 22;93(10):e00178-25. doi: 10.1128/iai.00178-25 (PMC12519781; doi:10.1128/iai.00178-25)
Supplement: Supplemental material — Fig. S1 to S5. [file iai.00178-25-s0001.pdf]

A)

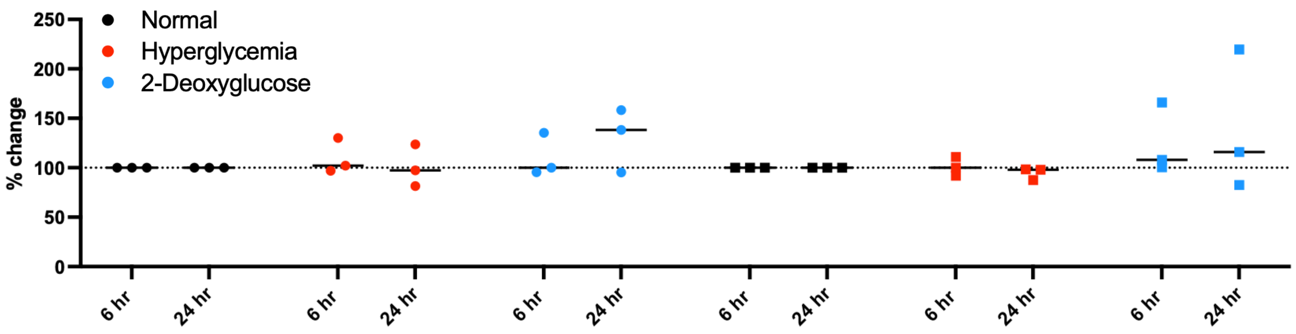

B)

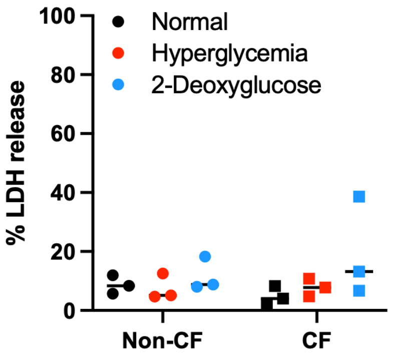

Supplemental Figure 1. Bronchial cells monolayer are not adversely affected by culturing at hyperglycemic level or with 2DG. A) Transepithelial electrical resistance (TEER) levels non-CF (circles) or CF (squares) cells. B) Lactate dehydrogenase (LDH) levels non-CF (circles) or CF (squares) cells. Data is reported as mean.

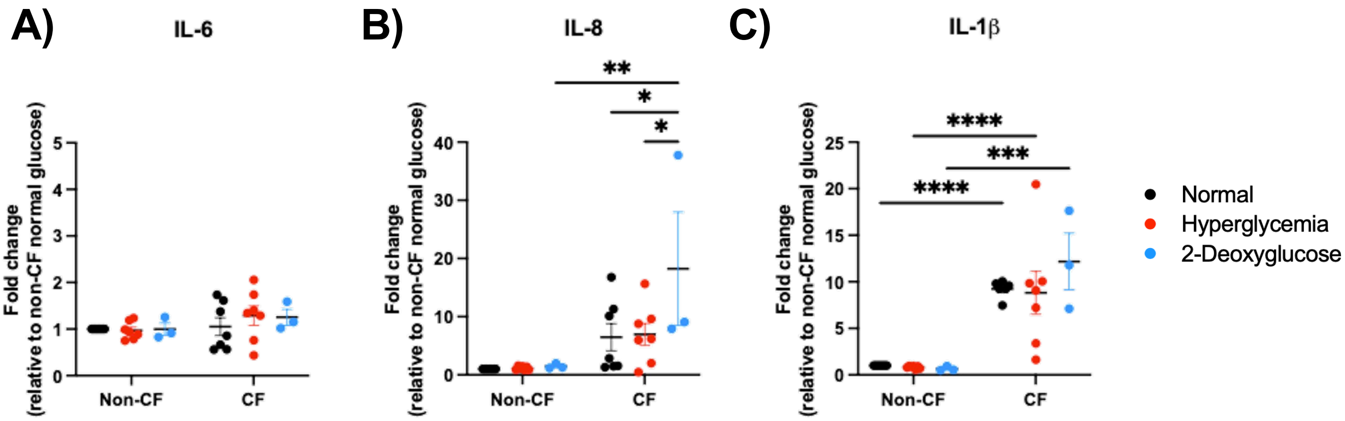

Supplemental Figure 2. Differential inflammatory cytokine expression in CF and non-CF bronchial epithelial cells maintained in normal and hyperglycemic conditions. A) IL-6 transcript levels B) IL-8 transcript levels & C) IL-1β transcript levels. Data reported as mean ± SEM. *P < 0.05, **P < 0.01, P < 0.001, ****P < 0.0001

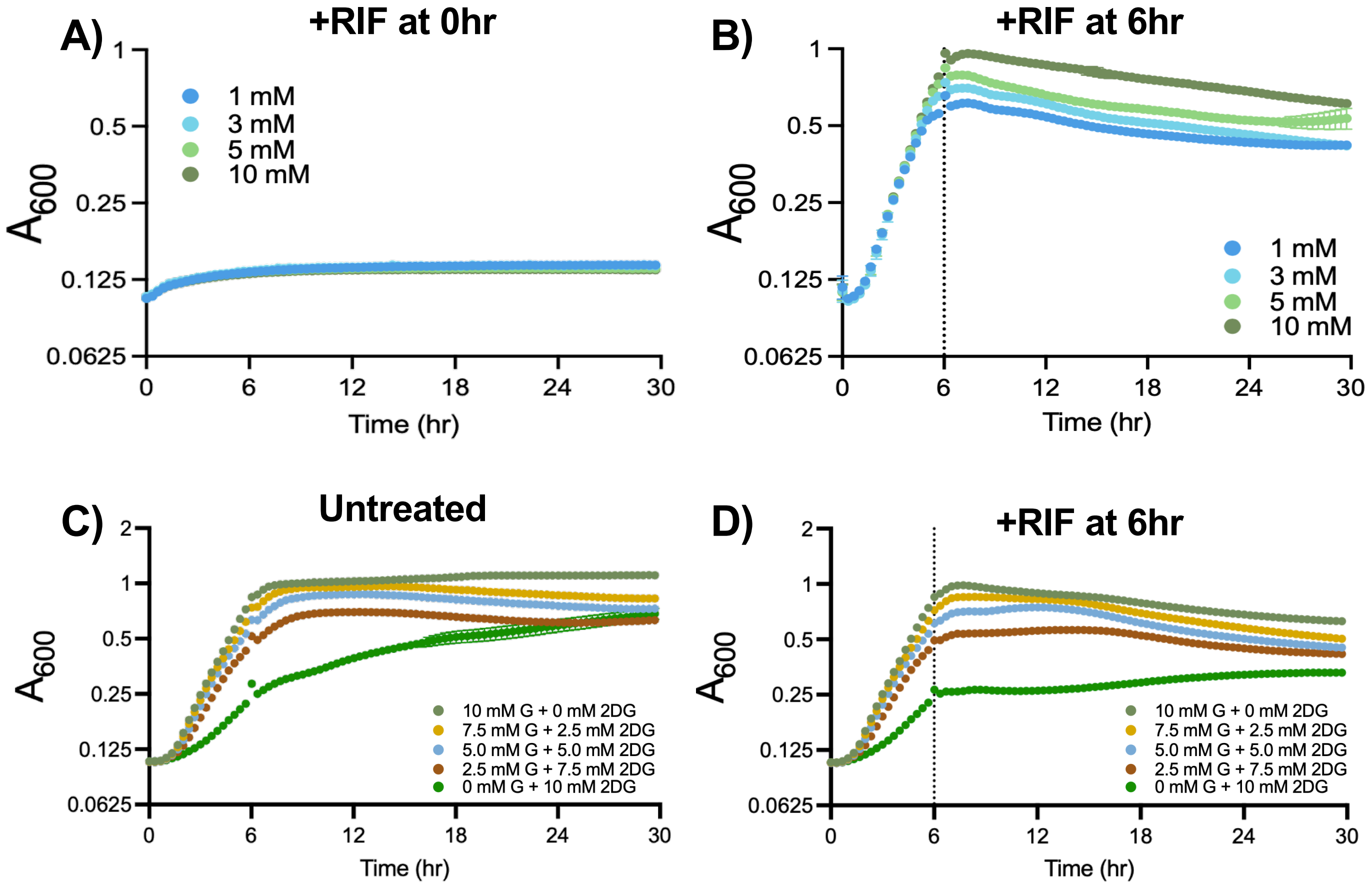

Supplemental Figure 3. Effects of rifampicin on growth of *S. aureus* in tryptic soy broth supplemented with glucose or 2-Deoxyglucose. A) Growth of *S. aureus* USA100 in tryptic soy broth (TSB) with rifampicin added at time of inoculation. B) Growth in TSB with rifampicin added after 6 hr of growth. Vertical dashed line indicates time of addition of antibiotic. C) Growth of *S. aureus* USA100 in TSB with glucose and 2-deoxyglucose (2DG) without rifampicin. D) Growth of *S. aureus* USA00 in TSB with glucose and 2DG with rifampicin added after 6 hr of growth. Vertical dashed line indicates time of addition of antibiotic. Data reported as mean ± SEM.

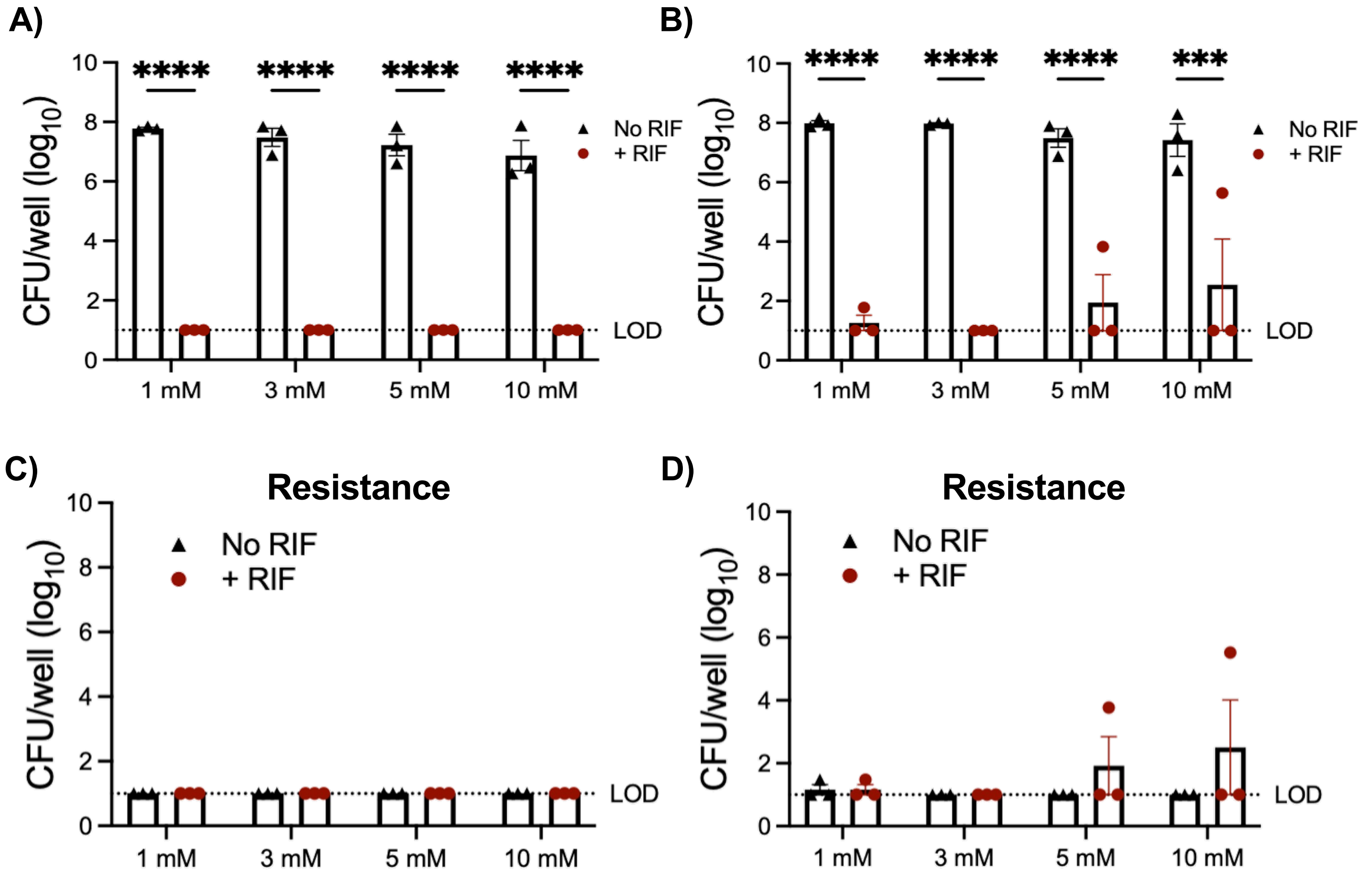

Supplemental Figure 4. *S. aureus* does not develop glucose-dependent rifampicin resistance in synthetic CF medium. A) CFUs of *S. aureus* from SCFM with or without rifampicin (RIF) added at the time of inoculum. B) CFUs of *S. aureus* from SCFM with or without RIF added after 6 hr of growth. C) CFUs of resistant *S. aureus* when grown together with RIF. D) CFUs of resistant *S. aureus* when RIF is added after 6 hr of *S. aureus* growth. Horizontal dashed lines indicated limit of detection (LOD). Data shown as mean ± SEM.

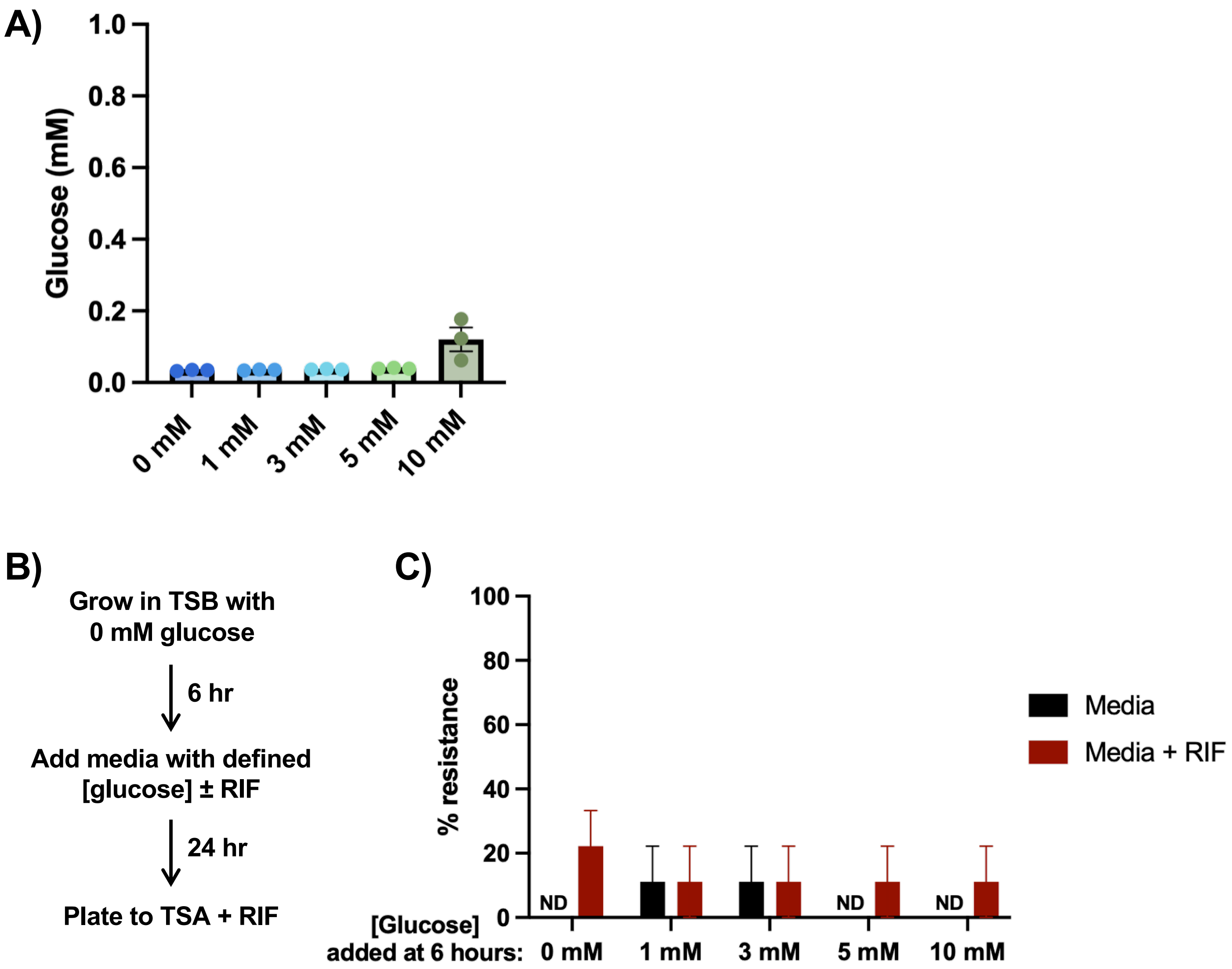

Supplemental Figure 5. Supplementation of cultures with glucose does not increase RIF resistance in TSB. A) Glucose concentrations measured in TSB after 6 hr of *S. aureus* growth show glucose is depleted at the time of RIF addition. B) Experimental setup for glucose supplementation in TSB culture to determine resistance outcomes. C) Percent resistance measured in *S. aureus* populations. N = 3 with three technical replicates per condition. Conditions in which no resistance was observed via plating to RIF-containing media are noted as “not detected” (ND). Data shown as mean ± SEM.
